# Occupational hazards and bladder cancer—An umbrella review of the risk in workers exposed over the past 30 years

**DOI:** 10.3389/fpubh.2025.1667873

**Published:** 2025-10-06

**Authors:** Cristina Mandanach, Claudia Mariana Handra, Agripina Rascu, Sorina Hohor, Irina Luciana Gurzu, Marina Ruxandra Otelea, Alexandru Stefan Catalin Rascu

**Affiliations:** ^1^Doctoral School, University of Medicine and Pharmacy Carol Davila, Bucharest, Romania; ^2^Clinical Department 5, University of Medicine and Pharmacy Carol Davila, Bucharest, Romania; ^3^Clinic of Occupational Medicine, Colentina Clinical Hospital, Bucharest, Romania; ^4^Clinical Department 9, University of Medicine and Pharmacy Grigore T. Popa, Iaşi, Romania; ^5^Occupational Medicine Clinical Department, Clinical Recovery Hospital, Iaşi, Romania; ^6^Clinical Department 3, University of Medicine and Pharmacy Carol Davila, Bucharest, Romania; ^7^Urology Clinic, “Professor Doctor Theodor Burghele” Clinical Hospital, Bucharest, Romania

**Keywords:** bladder cancer, occupational carcinogens, occupational exposure, cancer risk, umbrella review

## Abstract

**Background:**

Occupational exposure accounts for the second preventable risk factor for bladder cancer (BC), after smoking.

**Objective:**

This study aimed to extract evidence-based data from the systematic reviews that included studies primarily from the past 30 years, based on a clearly defined method of exposure assessment.

**Methods:**

A literature search in PubMed, Web of Science, ScienceDirect, and Embase was conducted using variations of the keywords “BC,” “occupational exposure,” and/or “occupation.” The inclusion criteria for the umbrella review were the following: systematic reviews and meta-analyses focused on occupational exposure, with a clear definition of the exposure assessment, a risk estimate for BC, and a majority of data from 1993 to 2023. We did not include other forms of reviews or systematic reviews focused on the general population and environmental exposure. Data were synthesized based either on occupations or on occupational hazards. After the overlap checking, the remaining reviews were assessed for quality using AMSTAR 2 criteria and afterwards classified for high, moderate, or low evidence using the GRADE scale.

**Results:**

We found relevant associations with a high level of evidence for firefighters, hairdressers, painters, workers in the petroleum industry, and dry cleaners exposed to tetrachloroethylene. Concerning hazards, exposure to ortho-toluidine was also confirmed to be a risk by recent studies. Welders, veterans, and those exposed to hexavalent chromium at higher risk need more well-designed studies to be confirmed.

**Conclusion:**

Despite longstanding recognition of certain risks, occupational exposure remains insufficiently investigated. Recent data support the inclusion of occupations and hazards in the individual risk assessment for BC.

## 1 Introduction

Bladder cancer (BC) is the ninth most commonly diagnosed cancer in the world (1). The risk of developing BC increases with age, and over 90% of BC patients are older than 55 years of age (2). Men are 3–4 times more affected than women (3). This difference can be explained by the small number of women employed in the industries with exposure to BC carcinogens, the increased prevalence of smoking among men, and some protective effects in the female gender, like an older age at the menarche, late menopause, parity, and hormone replacement treatments with estrogen and progesterone. Although BC carcinogen is less common in female patients than in male subjects, women tend to have their first consultation much later than men and are often diagnosed at more advanced stages of cancer, resulting in a worse prognosis (4). This highlights the importance of defining the high-risk female population.

Results from epidemiological studies all around the world showed a large number of risk factors, usually classified as (5, 6): (a) constitutional (age, sex, ethnic, geographic, and reproductive factors), (b) genetics, (c) dietary, (d) medical conditions and treatments, (e) socio-economic, and (f) occupational and environmental.

The most common risk factor is smoking, which is responsible for 50%−65% of all cases of BC. The second important risk factor is the occupational exposure, which accounts, for example, for 6% in the UK statistics (7). Like many other occupation-related diseases, the prevalence is underestimated, and the awareness about this risk is insufficient. However, a 2016 review indicated that the percentage of BC patients who identified occupational exposure as a risk factor in a questionnaire was higher than the above-mentioned percentage (8).

In the new guide for microhematuria published in 2020 by the American Urological Association, the additional factors for an intermediate level of risk in a patient with microhematuria include occupational exposures to benzene chemicals or aromatic amines (e.g., rubber, petrochemicals, dyes). The lack of knowledge about the exposure (both in patients and doctors) could explain why other substances were not included in this guide. This umbrella review aims to assess the latest evidence about the occupational exposure to carcinogens and BC, which might be considered in the diagnosis algorithm or even in the post-exposure screening, together with other panels of biomarkers (9).

## 2 Methods

For this umbrella review, we followed the Preferred Reporting Items for Overviews of Reviews (PRIOR) guideline (10). The purpose was to identify all systematic reviews concerning occupational exposure that mentioned a defined estimation of the exposure, an estimation of the risk, or referred to occupational activities (Table 1). The reviews were selected only if they included articles concerning the exposure in the past 30 years. From a meta-analysis including a mixture of studies (some referring to older than 30 years and some more recent studies), we selected only the ones covering the period relevant for this update and re-performed the statistical analysis.

**Table 1:** Selection criteria.

**Domain**	**Inclusion criteria**	**Exclusion**	**Relevance for the review question**
Population	Reviews focusing on occupational exposure, with an explicit nominalization of an occupational domain, industry, or occupational hazard(s)	Reviews focusing on the general population, environmental exposure	Even if general population data may serve to support a causal relation, the occupational exposure has different characteristics of duration, intensity, and route of exposure, which might influence the risk
		Non-human	
Exposure	Any exposure measure was considered, but the estimation of exposure had to be defined; an occupationally well-defined population was also considered eligible in terms of exposure assessment if studies included in the review were based on a validated method of exposure assessment.	Reviews that did not include a critical appraisal of the exposure in the initial articles	Exposure assessment is critical for occupational-related diseases, particularly in multifactorial diseases, such as cancer. The scope of the review was to provide evidence for enhancing the diagnostic algorithm.
Outcome	A risk estimate of BC (incidence rate, OR, RR, SIR, and CI)	Other forms of cancer, without specific data on BC	From good quality reviews that mentioned the BC effect, only studies related to this relation were extracted
Review design	Systematic reviews with meta-analysis. We considered the following reporting criteria for evaluating whether a reference constituted a full systematic review [Krnic Martinic et al. (11)] •Review question •Reproducible search strategy (naming of databases, naming of search platforms/engines, search date, and complete search strategy) •Inclusion and exclusion criteria •Selection (screening) methods •Critically appraises and reports about the quality/risk of bias of the included studies •Information about data analysis and synthesis that allows the reproducibility of the results	Scoping reviews, pooled studies, non-systematic reviews, conference papers	Scoping reviews, pooled studies, non-systematic reviews, and conference papers do not fulfill the criteria
Timeframe	In the original articles, only/the majority of articles with exposure from the past 30 years (1993–2023) were considered	Reviews include only studies before 1993 or in which the conclusion is based on studies published before 1993	Occupational exposure is evolving in line with the technical and socio-economic context; in many countries, the exposure limit has been lowered, and companies will adopt better protection measures to reduce the risk
Language	English, Italian, Spanish, French		

A literature search was conducted using variations of keywords like “BC,” “occupational exposure,” and/or “occupation” through several databases: PubMed, Web of Science, ScienceDirect, and Embase for all systematic reviews and/or meta-analyses published before the end of 2023. This search was effective with the PUBMED database. A similar search for the other three databases was applied, but, as the automatic selection of systematic review or meta-analysis was not possible, a larger number of articles were retrieved. We kept all the articles found after these selections so as not to miss any important reviews. Initially, we identified 1,658 articles from the four databases. Articles were extracted by CM and were double-checked for errors by MRO. After removing 136 duplicates, the 1,522 publications remaining were screened by title and abstract by the authors. Many articles did not correspond to the initial criteria and were excluded. For those who fulfilled all selection criteria, the complete text of the articles was analyzed. If there were some uncertainties for the inclusion or the exclusion of the systematic review, a third expert in urology or in occupational medicine was consulted for clarification.

**Figure 1:**
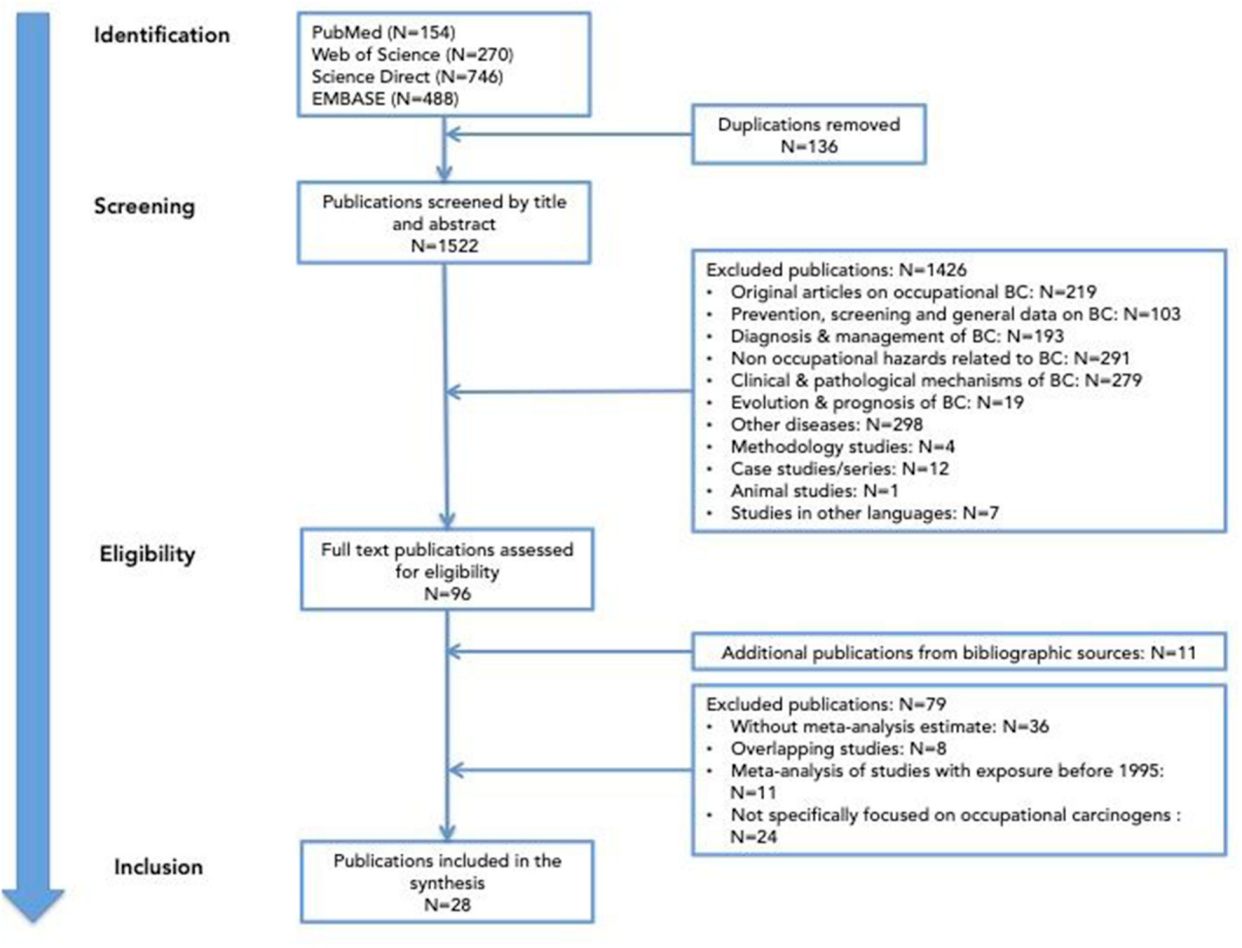
Literature flow diagram.

The flow of selection is presented in Figure 1.

The reviews selected that covered the same population or exposure were assessed for overlapping studies using the corrected covered area index (12). If the Corrected Covered Area index (CCAI) was >15%, the review, including more recent articles and/or a better quality, was presented. The quality of the systematic review was assessed based on the AMSTAR-2 guidance (13), independently, by two reviewers (CM and SH).

The following data were extracted from all studies: citation, review question, inclusion and exclusion criteria, risk of bias, heterogeneity, quality assessment, type of epidemiological study (e.g., retrospective cohort, case-control), number of subjects (exposed groups), carcinogen agent, period of exposure, follow-up period, occupational domain or worker type, exposure effect outcome estimates (e.g., standardized risk ratios, standardized mortality ratios, hazard ratio, odds ratios, risk ratios) with confidence intervals (CIs) and *p*-value, and additional details linked to the occupation, if available. The articles were analyzed by all authors and grouped by carcinogen, by occupational domain or job title, and their key findings (outcome measures) were summarized accordingly.

To assign the strengths of evidence in the comparison of these systematic reviews, the Grading of Recommendations Assessment, Development and Evaluation (GRADE) algorithm was applied by two independent reviewers (ILG and MRO) (14).

The review was registered in the Prospero register of systematic reviews (registration number CRD42025628025 (https://www.crd.york.ac.uk/PROSPERO/view/CRD42025628025).

## 3 Results

After applying all the selection criteria, 28 articles were analyzed. They were divided into two categories: (a) reviews focused on an occupational domain/occupation (17 articles) and (b) reviews focused on specific carcinogen(s) (11 articles). The plan to present the results corresponds to the IARC classification of hazards, which includes a list of agents (chemical, physical, and biological) as well as occupational processes where a mixture of carcinogenic agents is present. Another reason to synthesize the results in this manner is to facilitate the translation into clinical practice. At the end of the analysis, a summary of occupations/hazards for which the systematic reviews provide enough evidence is presented.

### 3.1 Systematic reviews focused on an occupational domain/occupation

Firefighters, painters, rubber manufacturing workers, hairdressers, military/veterans, agricultural workers, glass workers, motor vehicle drivers, petroleum industry workers, sales workers, and textile industry workers are the occupations that were subject to at least one systematic review (Supplementary Table S1).

A meta-analysis related to agricultural workers by Togawa et al. (15) included eight cohorts with an observation period of 31 years (1983–2014), with ≥15 years of enrolment in agriculture. The strengths of this meta-analysis include the large number of cohorts covering a wide geographic area, the presence of females among the workers, and the information on smoking and other confounders. Of note, people from none of the endemic areas for schistosomiasis were represented. A low risk of BC was found, but heterogeneity of exposure as type of farming (crop and livestock farmers, plant nursery workers, student farmers, and licensed pesticide applicators), active period (active or retired farmers), geographical location, and period of exposure, as well as different confounders, greatly limit the interpretation of this synthetic result.

Six systematic reviews [LeMasters et al. (16); Golka et al. (17); Jalilian et al. (18); Soteriades et al. (19); Casjens et al. (20); DeBono et al. (21)] were dedicated to firefighters' exposure to carcinogens. None was specifically designed for BC, but they collected data for different types of cancer. All analyses looked at the quality of the initial articles and the heterogeneity of results. These six publications include roughly the same studies, but three of them include exclusively (16) or mainly (17, 20) older studies. The meta-analyses finally included had a relatively high degree of overlap (16.67 and 11.54%). They also contained the studies from the publication of Golka et al. (17) and Casjens et al. (20), relevant for the time frame settled for this umbrella review (18, 19, 21). The incidence and mortality results for these three meta-analyses are similar and show a statistically significant increased risk. Soteriades et al. (19) estimated that the incidence and mortality were also calculated according to the quality of the studies. Thus, for good quality studies, these estimates are a bit higher—incidence 1.18 (95% CI 0.97–1.43), mortality 1.39 (95% CI 0.91–2.11), and incidence + mortality 1.24 (95% CI 0.98–1.57) (19). These meta-analyses have no smoking adjustment, but evidence suggests a reduced smoking prevalence among firefighters compared to the general population. Thus, smoking was not expected to be a statistically significant positive confounder. Also, some studies that also considered the smoking status indicated that the positive association persists after adjustment for smoking. The quality of these studies was moderate (19, 21) to high (18). Based on the above-mentioned results, it is most probable that the BC risk exists in firefighters, mainly after 10 years of exposure. The risk is significantly dependent on the respiratory protection used, making firefighters who have been recently exposed better protected when they have proper personal protective equipment and training.

Employees in the glass industry are exposed to numerous chemicals that belong to groups 1, 2, and 3 of carcinogens according to the IARC classification. The meta-analysis by Lehnert et al. (22) included a small number of studies, with a small number of BC cases, where three were included in the 1993 IARC report and two new ones. The oldest studies (23, 24) had the highest BC risk values: OR = 5.9 (95% CI: 0.7–49.8) and OR = 6.0 (95% CI: 0.7–276), respectively. In the only two newer case-control studies, the risk was statistically insignificant: Band et al. (25) OR = 1.51 (90% CI: 0.60–3.76) and Samanic et al. (26) OR = 1.10 (95% CI: 0.62–1.98).

Three publications concerned hairdressers, but one was excluded from the beginning, as it did not contain a meta-risk estimation. The other two (27, 28) had a high degree of overlap (42.59%). We selected the second because it presented data from two separate periods, among which the last one is relevant for our umbrella review. This second publication (28), was stratified by gender and working period: 1980–1989 RR = 1.24 (95% CI 1.00–1.53); after 1989 RR = 1.42 (95% CI 1.16–1.75); geographic location: for the USA and Canada RR = 1.28 (95% CI 1.08–1.52); for Europa RR = 1.34 (95% CI 1.19–1.52); quality of studies and smoking adjustment: adjusted for smoking RR = 1.35 (95% CI 1.13–1.61); no adjustment RR = 1.33 (95% CI 1.18–1.50) (28).

A first meta-analysis on peacekeeping forces exposed to depleted uranium, metals, and ultrafine particles (29) had a small number of cases and no concluding data on the interference with smoking habits. Overall, the quality of the studies included in the review did not justify a conclusion about a causal relation. The second meta-analysis included more studies and a much larger number of people (4,320,262 military veterans) (30). The degree of overlap was moderate (13.64%). Stratification of the results by type of exposure showed a higher risk, as expected, for depleted uranium exposure (30).

Motor vehicle drivers and BC risk were analyzed by Manju et al. (31). Most studies before 1990 showed a relation with the duration of exposure; research conducted after 1990 showed a slightly increased risk for BC, but lower than the one in previous periods (31), probably due to improvement in working conditions, but also in methodology (adjustment for smoking). In cohort studies, the pooled analysis risk was 1.08 (95% CI 1–1.17) with borderline significance.

The International Agency for Research on Cancer dedicated a monograph to painters and the carcinogenic risk (32). The meta-analysis presented in this monograph and the one conducted by Guha et al. (33) are concordant and consistent about the increase in risk, even after smoking adjustment, an expected result, as there is significant overlap of the articles included (CCAI = 50.79%). Bachand et al. (34) also reached the same results but had an even higher degree of overlap with the monograph (CCAI = 60%) and other meta-analyses (85.11%) and older studies (35, 36). What this meta-analysis adds is stratifying the risk according to the length of exposure and gender. Longer exposure (more than 10 years), female vs. male painters, and North America vs. European studies showed a higher risk (34). Even if there is a high rate of concordance between these meta-analyses, each of them has a high (32, 33) or moderate (34) quality evaluated by AMSTAR2. Based on these epidemiological data and exposure to a recognized class I carcinogen, the evidence was considered sufficient to include the painters in high-risk occupations.

The meta-analysis dedicated to the employees of the petroleum industry included a total of 190 incident cases of BC, with a significant effect size of 1.25 (CI: 1.09–1.43) (37). The incidence seems to be directly related to the intensity of hydrocarbon exposure, but this was mentioned in only one article containing data from exposure before 2000 (38). The mortality from BC was not associated with working in this industry. This decrease might result from the healthy worker effect or earlier diagnosis. Incidence studies that found a significant increase in BC risk were not adjusted for smoking, except for the initial analysis of an Australian cohort (39), where the relationship was independent of smoking. However, a 2-year later report of the same cohort did not confirm any increase in BC in this population (40). Based on these conflicting findings, a general conclusion cannot be reached, and only a case-by-case exposure assessment could validate the occupational influence.

Rubber manufacturing also benefited from an extensive literature review and one meta-analysis. The results were consistent for both case-control and cohort studies, but, as in the firefighters' case, they had a geographical influence (41).

The sales workforce was analyzed in-depth in a systematic review, showing a high publication bias and no differentiation based on the specific industry where the salespersons worked (42), which might be responsible for different exposures. For example, salespersons might have an exclusive office job or spend their time driving. Furthermore, the lack of a pathological mechanism of this association makes the results and the connection less probable.

BC risk analysis for workers in the textile industry was carried out on different types of exposure—cotton fibers, synthetic fibers, and wool fibers, among which none had a statistically significant result (43).

In a systematic review by Welders (44), only two studies showed a significant association with BC. Puntoni et al. found, in a mortality study of a cohort of shipyard workers, a RR = 2.74 (95% CI 1.02–7.35) for BC in welders using the electric arc procedure (45). In the second study, of 12,845 welders, the risk was elevated only for those with prolonged exposure, with RR = 1.40 (CI 95%1.15–1.70) (46). Adjustment to smoking was not performed. The quality of the meta-analysis was excellent (the GRADE score was high). The authors' conclusion about this association is that this might not be conclusive, as confounders were not taken into account, and the intensity of the exposure has not been assessed.

### 3.2 Systematic reviews focused on specific carcinogen(s)

For each of the following: arsenic, asbestos, benzene, bitumen, cobalt, diesel exhaust, hexavalent chromium, ortho-toluidine, tetrachlorethylene, and welding fumes, we found one meta-analysis. Pesticides benefited from two meta-analyses (Supplementary Table S2), which included totally different articles without any overlap.

Arsenic exposure from various industries and agriculture was examined based on the assumption that environmental exposure increases the risk of genitourinary cancers (47). Most of the studies included in Sassano et al.'s meta-analysis (11/17 studies) (48) provided data on BC. The risk was statistically insignificant for studies after 2000, RR = 0.94 (CI 0.68–1.32, *I*^2^ = 73%).

Asbestos has been banned in many countries since 1992, although certain exposure persists, particularly for the construction workers. Therefore, data on exposure are beyond the proposed timeframe. The reason we kept the systematic reviews focused on asbestos is by analogy with the very long latency period described for other cancer type localizations (49). Two systematic reviews were retrieved; one of them (50) included only old cohorts and was excluded. The second (51) covered cohorts from Europe, the UK, the US, Canada, Australia, and Asia in three different periods of employment: 1908–1940, 1941–1949, and 1950–1993. Overall, the incidence studies showed a decreased risk in the most recent periods of exposure. One study better reflected the exposure during the period selected in this review (52), showing no statistically significant risk for BC, SMR = 0.42 (CI 95% 0.03–6.41).

Benzene and BC risk were dose-related, and even job exposure matrices have been developed for this risk in the US (53). As many studies have been conducted in the chemical industry, exposure to PAH/other confounders (e.g., smoking) might be significant and could interfere with the results. Of note, for publications after 2000 from Seyyedsalehi et al.'s meta-analysis, a statistically significant risk was maintained, RR = 1.11 (95% CI 1.01–1.21), with a significant dose-dependent effect (54).

The review focused on bitumen exposure (55). Of all the 81 studies analyzed for the risk of cancer, only 13 cohorts, six case-controls, and four death certificate studies referred to BC. The exposure assessment was based only on occupation and/or industry, which was recognized as a limitation because some workers were probably not exposed (e.g., highway maintenance workers and/or operating engineers, landscaping workers, and crane operators each had limited bitumen exposure). The Nordic cohorts of asphalt workers showed some increase in risk after more than 30 years of employment in this job (56). Older cohorts had either a low number of cases (57), or contradictory results (58, 59). The case-control studies were more concordant for a higher risk (with four studies with a higher risk and only one European study with a lower risk). The meta-analysis of medium- and high-quality studies showed an m-ER = 1.03 (0.72–1.49) in the cohort and 1.03 (0.77–1.33) in case-control studies. Overall, the results do not support clear evidence about an increased risk of BC for workers exposed to bitumen.

To identify the relationship between cobalt and BC, seven occupational and 19 total joint replacement studies were included in a systematic review. The authors' hypothesis for selecting this combination of articles was that exposure in occupational settings possibly includes other carcinogens, while patients with hip prosthesis are exposed exclusively to cobalt-chromium alloy (60). This assumption is, however, doubtful, as patients might also work (or have worked) in workplaces with carcinogenic exposure or might have, in their home environment or lifestyle habits, such an exposure. From the seven studies focused on occupational exposure, only two (from Sweden and Finland) reported data on the incidence of BC in the time frame of our umbrella review. Results adjusted for smoking did not show a statistically significant value for BC risk: SIR = 1.02 (95% CI 0.64–1.55) (61). An excess of BC in workers with low exposure in the cobalt powder manufactory (SIR = 3.07; 95% CI 1.12–6.67) was reported, but 5/6 of the BC were in smokers (62). The final analysis stratified by different groups did not show any significant value to confirm that exposure to cobalt is a risk factor for BC. Cobalt is classified by the EU commission as a class IB carcinogen, and current epidemiological data are still insufficient to relate this agent to BC risk in the above-described occupations.

One meta-analysis referred to diesel exhaust, which found an increased risk, but also raised awareness about many limitations of the studies (63). The transportation domain seemed to be at a higher risk, SIR = 1.26 (95% CI 1.03–1.52) (64), while underground miners had a similar risk and altogether a lower than expected SMR (65). Publication bias was identified in the majority of the studies, and the conclusion was that the evidence of diesel exposure and BC is modest. There was only one cohort mortality study adjusted for smoking, and in this cohort, the RR was statistically insignificant (66). In the case-control studies, eight showed no increased risk, and four reflected a high risk either in bus or in truck drivers, particularly if the comparison was done between high exposure and low exposure.

For hexavalent chromium, the meta-analysis of Deng et al. (67) included 47 studies, of which 37 cohorts reported the SMRs and 16 the SIRs. The exposure times to Cr ^+VI^ were 1–10 years, 10–20 years, and over 20 years, the period for each study varying between 1985 and 2016. The follow-up periods were different for mortality: 5–10 years, 11–20 years, 21–30 years, 30–40 years, and 40–50-year; for cancer, which was grouped into three categories: 15–25 years (nine studies), 26–35 years (four studies), and 36–45 years (four studies). The study population consisted of over 1 million individuals from the USA, Europe (including the UK, Finland, France, Germany, Italy, Lithuania, Sweden, Switzerland, Iceland, Denmark, and Norway), and Asia (Korea and Japan). There were some positive results of the association with BC, but the analysis of more recent cohorts (closer to the selection criteria of our study) showed an insignificant increase in SMR (68, 69) or SIR (69, 70). Overall, the mortality studies showed an increased risk, while the incidence studies were statistically non-significant.

In the Report on Carcinogens Monograph on ortho-toluidine, six occupational historical cohorts, followed from 1946 to 2006, were analyzed (71). For the period included in this umbrella review, only three studies are relevant (72–74). Both the MR and the RR were maintained significantly, metaMR = 6.93 (95% CI 1.21, 39.54) and RR = 2.30 (95% CI 1.46–3.65), indicating that even in more recent cohorts, the risk is maintained.

The pesticide exposure was assessed for the green space workers in de Graaf et al.'s systematic review (75). The cohorts had a large geographical distribution, which influenced the risk. In most studies, the risk was lower than in the general population, particularly in the past 20 years, when limitation on exposure was implemented (76–78). In total, 10 studies found a decrease in the risk of BC in men and in women. From the more recent studies, e.g., cohorts of men from landscape and horticultural services in the USA who were followed up between 2001 and 2004, had a higher risk value of BC, expressed as OR = 2.4 (95% CI 1.2–4.8), with a positive trend in risk related to employment duration (79). Another review (80) found a statistically significant correlation between pesticide exposure and BC in the male group: OR = 1.144, (95% CI 1.076–1.217), in workers from America: OR = 1.741 (95% CI 1.270–2.388), and in the groups with exposure assessment extracted from the databases (pesticide or cancer register): OR = 1.148 (95% CI 1.079–1.221) or after adjustment for more than three confounding factors OR = 1.607 (95% CI 1.065–2.423). Two studies from the African region were included, with no analysis on the possible impact of schistosomiasis. The risk was significant in one cohort and statistically insignificant in the other. When stratified by study quality, high exposure to pesticides was associated with high risk of BC in both the low-quality group (five on the Newcastle–Ottawa Quality Assessment Scale) and the high-quality group (seven on the same scale), OR = 1.959 (95% CI 1.081–3.550), OR = 1.170 (95% CI 1.001–1.368), respectively. The different overall results of these two meta-analyses stem from the different studies included (0% of overlap). Both meta-analyses had a moderate level of quality for the evidence; however, the second included only older cohorts than our review time frame. Regarding case-control studies, there were more studies from the past 30 years than from previous periods. The GRADE score for both meta-analyses was moderate, with more biases in the initial articles and heterogeneity identified by de Graaf et al., and missing clear data on the databases used for the search of the articles by Liang et al. (80). Based on these findings, in terms of pesticide exposure, there are more arguments supporting it as a risk factor for BC with a moderate level of evidence.

For tetrachloroethylene exposure, we found a meta-analysis with a high-quality assessment of the exposure, but with few studies adjusted for smoking (81). This article (81) analyzed 14 case–control studies and five cohort studies, from the USA, Canada, Western Europe, New Zealand, Germany, Sweden, Denmark, Norway, and Finland. The overall mRR for BC in cohort studies was lower than the overall mRR among case-control studies. For the case–control studies that were adjusted for tobacco smoking, the mRR was similar to the mRR for the cohort studies, indicating that there is little evidence of confounding by tobacco smoking. A possible explanation for the higher mRR among the dry-cleaning workers would be co-exposure to an unidentified occupational bladder carcinogen or a lower exposure to tetrachloroethylene in laundry workers. RRs increased with the length of exposure and were the highest for those exposed for more than 10 years, RR = 1.57 (95% CI 1.07–2.29) (81).

For practical application, a list of the main findings in the high-quality systematic reviews, together with proposed actions, is summarized in Table 2. For some already recognized class I carcinogens for which current data are inconclusive in high-quality systematic reviews and meta-analyses, more research is needed to substantiate public health actions and medical surveillance. Occupations and occupational hazards for which the level of evidence is not strong enough were not listed.

**Table 2:** Summary of the evidence.

**Type of effect**	**Level of evidence**	**Occupation/exposure**	**Suggested action**
High risk	High	Firefighters	Active screening
		Hairdressers	Active screening
		Painters	Active screening
		Petroleum industry	Active screening
		Exposure to ortho-toluidine	Active screening
		Exposure to tetrachloroethylene from the dry cleaners	Active screening
Possible high risk	High	Welders	Active surveillance
	High	Veterans	Screening for exposure to depleted uranium For the rest, active surveillance
	High	Exposure to the hexavalent chromium	Active surveillance
Low risk based on current data	High	Arsenic exposure in agriculture	More research needed
	High	Bitumen	More research needed

## 4 Discussion

The carcinogens are currently classified by international or national regulatory bodies in different ways and according to distinctive criteria. Despite these differences, there is a certain overlap in what concerns the majority of them. IARC includes in the list of agents with “sufficient evidence in humans as BC carcinogens.” Five chemical substances (4-aminobiphenyl, arsenic and inorganic arsenic compounds, benzidine, 2-naphthylamine, and ortho-toluidine), six occupational activities/occupations (aluminum production, auramine production, magenta production, rubber manufacturing industry, working as a firefighter or working as a painter), two antineoplastic drugs (chlornaphazine and cyclophosphamide), two addictive behaviors (tobacco smoking and opium consumption), one infection (*Schistosoma haematobium*), and two physical agents (X-ray and gamma-radiation) (82). The CE list is not based on target organs but includes the class IA carcinogens, meaning the ones for which there is sufficient evidence in epidemiological studies in humans (83). The class IB list includes carcinogens with sufficient evidence in animal studies but insufficient in humans; both classes require special prevention measures. All five chemical substances mentioned above are listed as class IA on the CE list and as bladder carcinogens in the Agency for Toxic Substances and Disease Registry (ATSDR) list of the US (84). As research clarifies their status, several new studies have been conducted on these hazards. All these hazards should be subject to precautionary measures and workplace risk assessment. But as working conditions continue to change, an update of the more recent data is needed. We retrieved only data about arsenic, 2-naphthylamine (and possible mixture exposure with benzidine), and ortho-toluidine, which confirmed the tendency of mitigation of the risk in persons more recently employed, most probably from better general and personal protection measures.

Our first results referred to the risk of BC according to occupation. Firefighters, painters, and workers from the petroleum industry also encountered a higher risk, even in more recent studies included in high-quality reviews. A relation with the duration of exposure was found for firefighters (using a threshold of 10 years of exposure), but not for painters. The latest findings could be attributed to variable exposure to substances such as 4-aminobiphenyl or naphthylamine, which have no known carcinogenic threshold of exposure (85). This result implies that, under the current evidence, it is impossible to determine the level of exposure that does not increase the risk for cancer. Notably, for those few systematic reviews that had sufficient data to analyze the trend in incidence related to the updated protective measures introduced in different domains, a mitigation of the risk has been noticed for firefighters. This is also the case for workers' exposure to ortho-toluidine and for asphalters. Despite the reduction in cases in these occupations, a comprehensive occupational history is still necessary to exclude individual risk.

We could compare our results on occupations with a meta-analysis published in 2008 by Reulen et al. (86). This meta-analysis estimated that six occupations (hairdressers, painters, blacksmiths and tool-makers, dye makers, leather workers, and metal processors) had a positive association with BC from studies conducted after 1990. Concerning hairdressers, the only systematic review that focused exactly on the period of time selected for this umbrella review (from 1995 to 2021) (87) had a low degree of overlap with the previous ones (2.30%). In this systematic review, only four studies were included: three from Europe, in which no statistically significant result was found, and one case-control study from New Zealand, with nine times the odds of BC in hairdressers vs. the general population. However, these odds were based on a small number of cases, and the meta-risk estimate was not calculated. These differences can be explained by the fact that, since 2009, several carcinogens have been restricted in cosmetic products in the EU (88); thus, the risk has been significantly lower in European workers compared to those from other continents (87). The epidemiological data are supported by recent human cell studies, which showed the genotoxic effect of dyes on the urothelial mucosa (89) and a positive micronuclei test after exposure (90). Therefore, at least in countries where there is no limitation on exposure, workers have to be considered at risk.

Compared to this meta-analysis, four more studies referred to painters in the time frame of our research (77, 91–93). Three of them were case-control studies and estimated an OR from 1.7 to 3.01 (91–93). The fourth is a detailed record-linked study from the European Nordic countries; although the SIR was 1.08 (CI 95% 1.03–1.14), painters were not the workers with the highest risk (77). Other occupations (waiters, working in the tobacco industry) had higher odds of BC, but in these cases, a significant influence of smoking was mentioned. This cohort from the Nordic Countries, although comprising a large number of data points, may not be representative of the painting industry in other countries. Therefore, the close surveillance and active screening of those exposed to high levels (or with a long time of exposure) is still justified. For the petroleum industry, the difference is probably explained by the level of overlap, which was very low (3.57%). The new meta-analysis included more recent studies (10 out of 13 matched the time frame of our research). The Reulen's study found an OR = 1.15 (0.97–1.36), but in two articles of the ones that strictly referred to our time frame, the risk was higher in one (94) and statistically non-significant in the other (40). Some occupations did not benefit from systematic reviews in the last 30 years (leather workers, blacksmiths). We also did not retrieve any studies published more recently than 2008 concerning the association between BC and these occupations. The ones that are included in Reulen's meta-analysis found either an increased (95) or a decreased SMR (96) in leather workers. Research on blacksmiths included in this meta-analysis is predominantly before 1980, with no article containing data after 2000.

Our second line of results pertains to BC risk after exposure to specific occupational hazards. We found rather convincing data about the relevant timeframe, occupational exposure, and ortho-toluidine, tetrachloroethylene, and the BC risk. As previously mentioned, ortho-toluidine, an aromatic amine, is used in the rubber industry, in dyes, pesticides, and chemical manufacturing, and is already on the IARC list. Our data confirm that even in more recent exposures, the risk remained significant.

Tetrachloroethylene and dry cleaning occupation were classified by IARC, based on the experts' evaluation in 2012, as a probable carcinogen (group 2A) (97). The meta-analysis included in this umbrella review was published after the IARC monograph and adheres to the methodology of the agency, providing epidemiological evidence of an increased risk among dry cleaners. However, according to this evaluation, a dose-exposure relation was not sufficiently proven. In Reulen's meta-analysis, launderers had an RR for BC of 1.37 (95%CI 0.67–2.79) in studies published after 1990. There was a high overlap between Reuelen's meta-analysis and ours (CCAI = 33.3%), which primarily stemmed from studies conducted before our research purpose. These differences in exposure periods might explain the difference. To the best of our knowledge there is only one more recent study on this topic, which analyzed mortality in 22 years of follow-up of a cohort of 5,369 dry cleaning employees from the U.S. They found a dose-relation response for BC: HR 4.2, 95% CI 0.7–24.5 and 9.2, 95% CI 1.1–76.7 for medium and high exposure, respectively vs. no exposure (98). Therefore, as a precautionary measure, patients working as dry cleaners, with high exposure, should be included in the category of high risk.

There are also exposures in which the clear-cut between occupational and non-occupational exposure is even more difficult to define. Cobalt, arsenic, and pesticides would fit in this category. For those, the occupation, industry, and even workplace data might not be enough. The history must be completed with more data on lifestyle, hobbies, home exposure, or exposure biomarkers. In particular, for pesticides, the current data are not enough, and further studies are needed.

Some carcinogens (such as chromium, arsenic, cobalt, and diesel exhaust) have been linked with other forms of cancer, and the existing data about their BC causality are controversial. They would need more studies, particularly focused on identifying the BC risk, with better exposure assessment. Until then, a case-by-case assessment is probably the reasonable approach.

The present umbrella review reflects the methodological strengths and weaknesses of the systematic reviews it includes, particularly in terms of quality and design. There are some limitations in the systematic reviews synthesized here, some of which are inherently difficult to avoid. Most of the limitations come from the challenges in accurately estimating the exposure. These include variability over time, differences in industrial processes across workplaces, inconsistent use of protective measures, and the lack of data on non-occupational exposures, which are often unmonitored or poorly characterized outside occupational settings. Some of these systematic reviews failed to collect methodologically robust studies or reported major concerns about the assessment of exposure. For example, the cadmium (99) or radon systematic reviews (100). Although it suggested a higher risk, it did not summarize the results in a synthetic risk estimate and did not fulfill our inclusion criteria. In certain cases, the non-occupational exposure might be more relevant than the occupational one. Cohort studies based on national registries of mortality do not have detailed data on the exposure; the name of the occupation gives only a very rough estimation of the exposure, and the cumulative exposure has a certain degree of uncertainty. For agents with no known threshold level for carcinogenicity, this cumulative exposure may be less informative; however, the duration of exposure and, consequently, the likelihood of direct contact and absorption, still contribute to the risk. A certain influence on the results stems from the inclusion of older cohorts, despite our selection of the meta-analysis based on more recent data. It was not the scope of this review to calculate the risk from these newly published data. For each of our findings, we have looked for other references to confirm the results. Finally, BC is less common than other cancers, such as lung cancer, which makes it more difficult to gather large enough cohorts to achieve statistically robust conclusions. Addressing these limitations requires cautious interpretation of the findings, which supports the need for improved individual-level exposure data in future research.

Despite these limitations, there are some practical implications. Based on the current evidence, certain occupational groups should be considered for targeted screening and long-term medical surveillance due to their increased risk of BC associated with workplace exposures. The list includes firefighters, painters, workers in the petroleum industry, dry cleaners, hairdressers, and those exposed to ortho-toluidine with long-term employment in these occupations. Depending on detailed occupational histories, other groups such as welders, veterans, and workers exposed to chromium may also warrant further evaluation. Systematically integrating occupational exposure history into clinical algorithms for hematuria assessment and cancer risk stratification could enhance early detection, particularly among high-risk populations of workers. An update of this type of actualized information can be utilized in health policy and screening programs, which can lead to earlier diagnosis and, consequently, to achieving effective and visible results by decreasing the incidence and mortality rates of BC. Public health authorities and guideline developers should consider incorporating post-exposure monitoring protocols and tailored risk communication strategies that reflect occupational contexts.

## 5 Conclusion

Occupational exposure has to be included in the risk assessment of suspected BC cases. There is sufficient evidence for a high risk associated with all class I carcinogens; the length, intensity, and context of exposure should be evaluated despite continuous efforts to improve workers' protection and the reduction in the number of cases. Recent data provide arguments for an increased risk in firefighters, painters, and workers from the petroleum industry, or those who are exposed to ortho-toluidine and tetrachlorethylene, particularly dry cleaners. Regional or national regulations and industry practices should be considered, as they might significantly influence exposure levels and associated risks.

Despite longstanding recognition of certain risks, occupational exposure remains insufficiently investigated. Given the long latency period between exposure and disease onset, medical surveillance should continue even after a worker leaves the job. While some historical exposures may be declining due to effective protection policies, new or evolving occupational settings may introduce risks that are currently underestimated or undocumented, requiring proactive research and regulatory attention.
