# Supplementary material for: Occupational hazards and bladder cancer—An umbrella review of the risk in workers exposed over the past 30 years
Source: Front Public Health. 2025 Oct 6;13:1667873. doi: 10.3389/fpubh.2025.1667873 (PMC12536731; doi:10.3389/fpubh.2025.1667873)
Supplement: Supplementary file 1 [file Table_1.docx]

Supplementary Material

**Supplementary Table 1. Occupational domain and bladder cancer risk**

| Domain/ occupation | Occupational hazard | Number of studies | Main results | AMSTAR 2 grading | GRADE level of evidence | Reference |
| --- | --- | --- | --- | --- | --- | --- |
| 1.Farmers/ agricultural workers | Pesticides, solar radiation | 8 cohorts | m-SIR = 0.62 (95% CI 0.56–0.68),  men m-SIR = 0.63 (95% CI 0.55-0.72)  women m-SIR = 0.63 (95% CI 0.47-0.84) | Low | Moderate | Togawa K, 2021 (15) |
| 2.Firefighters | Aromatic amines and PAHs, combustion products (soot), heavy metals (cadmium and arsenic), diesel exhaust, heat. | 4 case control (registry based), 9 cohorts;  6 cohort studies included in the mortality risk estimation | SIRE for BC = 1.12 (95% CI 1.04-1.21);  SMRE = 1.22 (95% CI 0.93-1.6) | High | High | Jalilian H, 2019 (18) |
| 3.Firefighters | Aromatic amines and PAHs, combustion products (soot), heavy metals (cadmium and arsenic), diesel exhaust, heat. | 49 studies: 26 cohort studies, 17 case-control and 6 surveillance studies | Incidence RE = 1.06 (95% Cl 0.88 – 1.27),  Mortality RE = 1.28 (95% CI 1.05–1.56),  Incidence + mortality RE = 1.18 (95% CI 1.01–1.36) | Moderate | Moderate | Soteriades ES, 2019 (19) |
| 4.Firefighters | Aromatic amines and PAHs, combustion products (soot), heavy metals (cadmium and arsenic), diesel exhaust, heat | 10 cohort studies | Incidence mRR = 1.16 (95% CI 1.08-1.26) Mortality mRR = 1.22 (95% CI 0.70–2.11); | High | Moderate | DeBono N L, 2023 (21) |
| 5.Glass workers | Silica dust, metals, asbestos, polycyclic aromatic hydrocarbons, dyes | 5 studies for BC, only 2 after 1995 | mOR = 2.09 (95% Cl 0.79-5.53) | Low | Moderate | Lehnert M, 2020 (22) |
| 6. Hairdressers | Hair products, chemicals contained in hair dyes | 42 studies | RR = 1.34 (95% Cl 1.21-1.48),  Women RR = 1.25 (95% Cl 1.05-1.50,  Men RR = 1.52 (95% Cl 1.34-1.72),  Employment >5 years RR = 1.52 (95% CI 0.79-2.93);  Employment >10 years RR = 1.70 (95% CI 1.01-2.88) | High | High | Harling M, 2010 (28) |
| 7.Military service/ veterans | Uranium, metals, and ultrafine particles; aromatic amines, diesel exhaust – no clear exposure in these circumstances. | 3 cohort studies | Meta-estimate of risk of BC was increased two-fold  Fixed effect summary [FES] = 2.16 (95% CI 1.35 – 2.97). | Low | Low | Cocco P, 2022 (29) |
| 8.Military service/ veterans | Agent Orange, depleted uranium, contaminated water | 4 studies Agent Orange, 4 studies depleted uranium,  2 studies for contaminated water with perfluoroalkyl, substances volatile organic compounds, trichloroethylene, perchloroethylene | Agent Orange HR = 1.17 (95% CI 1.01-1.36).  Depleted uranium HR = 2.13 (95% CI 1.31-3.48),  Contaminated water HR = 1.25 (95% CI 0.97-1.61) |  | High (only for depleted uranium) | Kronstedt S, 2024 (30) |
| 9.Motor vehicle drivers | Air pollutants, such as polycyclic aromatic hydrocarbons, diesel exhaust | 3 cohort studies and 27 case control studies; stratification by period: 1998-2008 and 1977-1987 | For studies published 1998-2008, the pooled risk:  truck drivers: 1.20 (95% CI 1.00-1.40);  bus drivers 1.21 (95% CI 0.72-2.01);  railroad workers 1.25 (95% CI 0.96-1.61);  cohort studies the pooled risk for all occupations 1.08 (95% CI 1.0-1.17) | Moderate | Moderate | Manju L, 2009 (31) |
| 10.Painters | Metal coating, wood varnishes or stains, solvents | 30 case-control studies and 11 cohort and record linkage studies | Meta-RR, (random effects) = 1.25 (95% CI 1.16-1.34)  Higher RR if exposure is longer than 10 years (m-RR=1.81) compared to less than 10 years (m-RR =1.41). | High | High | Guha N, 2010 (33) |
| 11.Painters | Pigments, extenders, binders, stabilizers, resins, organic and mineral solvents, additives in paints, dusts (possibly asbestos, crystalline silica) and fumes; non-paint compounds such as paint strippers, cleaning agents | 40 case control  9 cohorts for mortality  4 cohorts for morbidity | Case control (unadjusted) 1.28 (95% CI 1.7-1.41)  Case control smoking adjusted OR =1.3 (95%CI 1.17-1.44)  Morbidity (unadjusted) cohort studies: RE= 1.14 (95% CI 1.06-1.22)  Cohort studies morbidity external adjustment for smoking RE 1 1.23 (95% CI 0.71-1.27) | Moderate | High | Bachand A, 2010 (34) |
| 12.Painters | Dyes, metals (cadmium, chromium hexavalent), formaldehyde, aromatic hydrocarbons, nickel, rubber synthetic | 11 cohort and record linkage studies and 28 case–control studies | Meta-RR cohort studies = 1.21(95% CI 1.11–1.32);  Meta OR case control studies = 1.28 (95% CI 1.16–1.43);  Combined RR: meta-RR= 1.24 (95% CI 1.16–1.33);  Risks higher in women (meta-RR, 1.54 (95% CI 1.03–2.31) than in men (meta-RR, 1.27 (95% CI 1.18–1.36).  Risks higher in North America (meta-RR, 1.32 (95% CI 1.20–1.46); I2 = 0%, P = 0.73) and Europe (meta-RR, 1.19 (95% CI 1.08–1.31); I2 = 24.1%, P = 0.18). | High | High | IARC Painting, Firefighting, and Shiftwork, 2010 (32) |
| 13.Petroleum industry | Heavy metals and hydrocarbons such as benzene, benzidine, asbestos, crude oil | 13 cohort studies on BC  9 mortality studies and  6 incidence studies | Pooled effect size Incidence =1.25 (95% Cl I 1.09-1.43)  Pooled effect size mortality 0.91 (95% CI 0.79-1.04) | High | High | Onyije FM, 2021 (37) |
| 14.Rubber manufactury | 1,3-butadiene and benzene | 54 studies (35 cohort and 19 case control) | SRR =1.36 (95% CI 1.18-1.57);  SRR higher in women: 2.23 (95% CI 1.39-3.60) than men (SRR= 1.26 (95% CI 1.09-1.45);  Workers hired after 1970 SRR = 0.81 (95% CI 0.40-1.64) | Low | Low | Boniol M, 2017 (41) |
| 15.Sales occupations | Carcinognes from some sectors of sales work, engine exhaust | 15 studies, of which 10 cases control | Overall: men OR 1.04 (95% CI 0.97–1.12)  women OR 1.22 (95% CI 1.06–1.41)  Incidence: men OR 1.07 (95% CI 0.99-1.15)  women OR 4.78 (95% CI 1.45–15.8) | Critically low | Low | 't Mannetje A, 2006 (42) |
| 16.Textile industry | Different type of fibers, dyes | Studies from IARC monograph, 1990 and 4 additional studies | Carders and fiber preparers PRR=1.50 (0.55 -3.26) - 1 study;  Pinners and weavers PRR= 1.19 (0.80-1.57); Weavers PRR=2.40 (1.62-3.18), p<0,05;  Dyers PRR=1.39 (1.07-1.71), p<0,05 | Low | Low | Mastrangelo G, 2002 (43) |
| 17.Welding fumes | Welders | 7 studies | RR=1.26 (95% CI 0.98–1.60) | High | High | Collatuzzo G, 2024 (44) |

**Supplementary Table 2. Carcinogen(s) and bladder cancer risk**

| Carcinogen | Domain/  occupation | Number of studies | Main results | AMSTAR 2 grading | GRADE level of evidence | Reference |
| --- | --- | --- | --- | --- | --- | --- |
| 1. Asbestos | Asbestos workers, miners and millers, and shipyards | Cohorts with workers employed from 1950–1993;  10 studies on mortality and  8 studies on incidence | SMR=1.00 (0.75–1.33)  SIR= 0.90 (0.77–1.06) | High | High | Franco N , 2023 (51) |
| 2.Arsenic | Smeltery, chemical and petrochemical, agriculture, insecticide production, tin or gold mine and refinery of metals, uranium mine, copper smelters | 11 studies referring to BC; 3 on incidence and 8 on mortality data | Incidence studies showed RR= 1.26 (95%CI 0.89-1.80)  Mortality studies showed RR= 0.73 (95% CI 0.50-1.07) | High | High | Sassano M, 2023 (48) |
| 3.Benzene | Petroleum industry, shoemakers,  paint production and painters, chemical industry, rubber industry, printing,  and laboratory workers. | 41 studies, (33 cohort and 8 case-control) | RR=1.07 (95%CI 0.97-1.18)  Case control: RR=1.17 (95%CI 0.95-1.45)  Cohort RR=1.04 (95%CI 0.92-1.19)  Publications after 2000, the RR=1.11 (95%CI 1.01-1.21) | High | Moderate | Seyyedsalehi MS, 2025 (54) |
| 4.Bitumen | Bitumen- exposed workers (roofers, pavers) | 13 cohort studies  4 death certificate studies  6 case-control studies for bladder cancer | Meta-RR=1.09 (95% CI 0.93-1.27), for cohort studies  Meta-RR =1.01 (95% CI 0.87-1.18) for death certificate studies  Meta-RR = 1.66 (95% CI 1.00-2.74) for case-control studies | Moderate | High | Mundt KA, 2018 (55) |
| 5.Cobalt | Metal plants, steel factory, cobalt plant, porcelain factories, hard metal production plant | 7 occupational exposure studies | Occupational exposure (overall): REE= 0.74 (95% CI 0.22–2.56)  Exposure for 5-10 years: REE=0.98 (95% CI 0.92–1.05)  Exposure for >10years: REE=0.81 (95% CI 0.42–1.57) | Hgh | Moderate | Holy CE, 2021 (60) |
| 6.Diesel exhaust | Drivers, railroad workers, bus maintenance workers, operators of heavy machines in ground and road construction | 35 studies, 14 on railroad workers and 15 on truck drivers and 10 on bus drivers;  7 cohort studies and 2 incidence studies | Truck drivers RR= 1.17 (95% CI 1.06 –1.29)  Bus drivers RR=1.33 (95% CI 1.22–1.45)  Studies considering diesel exhaust in the JEM: RR1.44 (95%CI 1.18-1.76) | Moderate | Moderate | Boffetta P, 2001 (63) |
| 7.Hexavalent chromium | Workers in chromate production, cement industry; stainless steel welders, chrome platers, aircraft manufacturing workers, tanners, painters, and masons; separate analysis performed for regional studies, gender, occupations (welders, tanners, steel production) | 37 cohorts with mortality reported and 16 with incidence reported from 47 separate studies. | Meta-SMR was 1.24 (95% CI 1.05–1.47), The SIR was not statistically significant for neither of the variables analyzed. | High | High | Deng Y, 2019 (67) |
| 8.Ortho-toluidine | Rubber chemical industry, dyestuffs industry and in plant manufacturing 4-chloro-ortho-toluidine | 6 occupational historical cohort studies of cancer and 1 population-based case-control study | Rubber chemical workers SIR = 3.90; 95% CI 2.57-5.68  U.K. rubber chemical cohort study SIR = 5.56 (95% CI 1.51-14.22) and SMR = 11.2 (95% CI 2.30-32.61),  Italy dye workers SMR = 22.5 (95% Cl 8.3–49.0) UK magenta production SMR = 23.0 (95% CI 5.87–62.8) | High | High | Report on Carcinogens Monograph on ortho-Toluidine RoC Monograph 04, 2014 (71) |
| 9.Pesticide | Pesticide exposure | 7 case-control, 2 cohort | Overall: OR for BC =1.649 (95% CI 1.223-2.223).  For case-control OR=2.075 (95% CI 1.183-3.638)  For cohort groups OR=1.146 (95% CI 1.074-1.223) | Low | Moderate | Liang Z, 2016 (80) |
| 10.Pesticide | Greenspace workers | 15 cohort and 11 case-control | Ten studies found a decrease in the risk of bladder cancer for men SIR=0,8 [0,8-0,8]. In women, many studies showed a lower risk for bladder cancer SIR=0,8 [0,7-0,8] | Low | Moderate | de Graaf L, 2022 (75) |
| 11.Tetra-chloroethylene | Dry cleaning workers and laundry workers | 13 case–control studies, 11 cohort studies, 1 meta-analysis, and 1 cluster analysis | mRR= 1.08 (95% CI 0.82-1.42) for tetrachloroethylene-exposed workers,  mRR= 1.20 (95% CI 1.06-1.36) for laundry and/or dry-cleaning workers  mRR= 1.47 (95% CI 1.16-1.85) for dry-cleaning workers | Moderate | High | Vlaanderen J, 2014 (81) |

**Supplementary Table 3. Excluded articles**

| **Autor** | **Title** | **An** | **Link** |
| --- | --- | --- | --- |
| **Publication without meta-analysis estimate (36)** | | | |
| Ahmad, M. F | Pesticides Impacts on Human Health and the Environment with Their Mechanisms of Action and Possible Countermeasures | 2024 | https://doi.org/10.1016/j.heliyon.2024.e29128 |
| Al-Zalabani, A. H | Modifiable Risk Factors for the Prevention of Bladder Cancer: A Systematic Review of Meta-Analyses | 2016 | https://doi.org/10.1007/s10654-016-0138-6 |
| Alarcón-Capel, E | [Radon exposure and genitourinary cancer in miners] | 2021 | https://doi.org/10.1016/j.gaceta.2019.06.006 |
| Allen, E. M | Cancer Incidence among Minnesota Taconite Mining Industry Workers | 2015 | https://doi.org/10.1016/j.annepidem.2015.08.003 |
| Babić, Ž | Association of Hairdressing with Cancer and Reproductive Diseases: A Systematic Review | 2022 | https://doi.org/10.1002/1348-9585.12351 |
| Boffetta, P | Exposure to Permethrin and Cancer Risk: A Systematic Review | 2018 | https://doi.org/10.1080/10408444.2018.1439449 |
| Bosetti, C | Occupational Exposures to Polycyclic Aromatic Hydrocarbons, and Respiratory and Urinary Tract Cancers: A Quantitative Review to 2005 | 2007 | https://doi.org/10.1093/annonc/mdl172 |
| Carreón, T. | The Genetic and Environmental Factors Involved in Benzidine Metabolism and Bladder Carcinogenesis in Exposed Workers | 2006 | https://doi.org/10.2741/2017 |
| Carreón, T | Bladder Cancer Incidence among Workers Exposed to O-Toluidine, Aniline and Nitrobenzene at a Rubber Chemical Manufacturing Plant | 2014 | https://doi.org/10.1136/oemed-2013-101873 |
| Calvert, G. M | Cancer Risks among Workers Exposed to Metalworking Fluids: A Systematic Review | 1998 | https://doi.org/10.1002/(sici)1097-0274(199803)33:3<282::aid-ajim10>3.0.co;2-w |
| Czubacka, E | 2-Naphthylamine Toxicity | 2020 | https://doi.org/10.13075/mp.5893.00921 |
| Di Giovanni, P | Arsenic Exposure and Risk of Urothelial Cancer: Systematic Review and Meta-Analysis | 2020 | https://doi.org/10.3390/ijerph17093105 |
| Fanfani, A | Cadmium in Biological Samples and Site-Specific Cancer Risk and Mortality: A Systematic Review of Original Articles and Meta-Analyses | 2024 | https://doi.org/10.1016/j.canep.2024.102550 |
| Feki-Tounsi, M | Cadmium as a Possible Cause of Bladder Cancer: A Review of Accumulated Evidence | 2014 | https://doi.org/10.1007/s11356-014-2970-0 |
| Gibbs, G. W | Cancer Risks in Aluminum Reduction Plant Workers: A Review | 2014 | https://doi.org/10.1097/JOM.0000000000000003 |
| Guyton, K. Z | Human Health Effects of Tetrachloroethylene: Key Findings and Scientific Issues | 2014 | https://doi.org/10.1289/ehp.1307359 |
| Guzzo, T. J | Bladder Cancer and the Aluminium Industry: A Review | 2008 | https://doi.org/10.1111/j.1464-410X.2008.07903.x |
| He, L | Hair Dye Ingredients and Potential Health Risks from Exposure to Hair Dyeing | 2022 | https://doi.org/10.1021/acs.chemrestox.1c00427 |
| Jenkins, W. D | Population Cancer Risks Associated with Coal Mining: A Systematic Review | 2013 | https://doi.org/10.1371/journal.pone.0071312 |
| Kalan Farmanfarma, K | Bladder Cancer in Iran: An Epidemiological Review | 2020 | https://doi.org/10.2147/RRU.S232417 |
| Kogevinas, M | Cancer Risk in the Rubber Industry: A Review of the Recent Epidemiological Evidence | 1998 | https://doi.org/10.1136/oem.55.1.1 |
| Koutros, S | Diesel Exhaust and Bladder Cancer Risk by Pathologic Stage and Grade Subtypes | 2020 | https://doi.org/10.1016/j.envint.2019.105346 |
| La Vecchia, C | Cancer Risk in Carbon Electrode Workers: An Overview of Epidemiological Evidence | 2003 | https://doi.org/10.1097/00008469-200310000-00013 |
| MacLeod, J. S | Cancer Risks among Welders and Occasional Welders in a National Population-Based Cohort Study: Canadian Census Health and Environmental Cohort | 2017 | https://doi.org/10.1016/j.shaw.2016.12.001 |
| Mastrangelo, G | Polycyclic Aromatic Hydrocarbons and Cancer in Man | 1996 | https://doi.org/10.1289/ehp.961041166 |
| Mossanen, M | Exploring Exposure to Agent Orange and Increased Mortality Due to Bladder Cancer | 2017 | https://doi.org/10.1016/j.urolonc.2017.07.030 |
| Mundt, K. A | Critical Review of the Epidemiological Literature on Occupational Exposure to Perchloroethylene and Cancer | 2003 | https://doi.org/10.1007/s00420-003-0457-2 |
| Myong, J.-P | Overview of Occupational Cancer in Painters in Korea | 2018 | https://doi.org/10.1186/s40557-018-0222-3 |
| Pira, E | Bladder Cancer Mortality of Workers Exposed to Aromatic Amines: A 58-Year Follow-Up | 2010 | https://doi.org/10.1093/jnci/djq214. |
| Rota, M | Occupational Exposures to Polycyclic Aromatic Hydrocarbons and Respiratory and Urinary Tract Cancers: An Updated Systematic Review and a Meta-Analysis to 2014 | 2014 | https://doi.org/10.1007/s00204-014-1296-5 |
| Singh, Z | Textile Industry and Occupational Cancer | 2016 | https://doi.org/10.1186/s12995-016-0128-3 |
| Tagkas, C. F | Fertilizers and Human Health—A Systematic Review of the Epidemiological Evidence | 2024 | https://doi.org/10.3390/toxics12100694 |
| Vagnoni, G | Lead (Pb) in Biological Samples in Association with Cancer Risk and Mortality: A Systematic Literature Review | 2024 | https://doi.org/10.1016/j.canep.2024.102630 |
| Weiss, N | Cancer in Relation to Occupational Exposure to Perchloroethylene | 1995 | https://doi.org/10.1007/BF00051797 |
| Xie, S | Occupational Exposure to Organic Solvents and Risk of Bladder Cancer | 2024 | https://doi.org/10.1038/s41370-024-00651-4 |
| Xiong, X | An Umbrella Review of the Evidence Associating Occupational Carcinogens and Cancer Risk at 19 Anatomical Sites | 2024 | https://doi.org/10.1016/j.envpol.2024.123531 |
| **Overlapping studies (8)** | | | |
| Casjens, S | Cancer Risks of Firefighters: A Systematic Review and Meta-Analysis of Secular Trends and Region-Specific Differences | 2020 | https://doi.org/10.1007/s00420-020-01539-0 |
| Chen, R | A Meta-Analysis of Painting Exposure and Cancer Mortality | 1998 | https://doi.org/10.1046/j.1525-1500.1998.00a47.x |
| den Braver-Sewradj,S.P | Occupational Exposure to Hexavalent Chromium. Part II. Hazard Assessment of Carcinogenic Effects | 2021 | https://doi.org/10.1016/j.yrtph.2021.105045 |
| Golka, K | Fire Fighters, Combustion Products, and Urothelial Cancer | 2008 | https://doi.org/10.1080/10937400701600396 |
| IARC | Occupational exposure as a painter | 2012 | https://www.ncbi.nlm.nih.gov/books/NBK304433/ |
| Laroche, E | Cancer Incidence and Mortality among Firefighters: An Overview of Epidemiologic Systematic Reviews | 2021 | https://doi.org/10.3390/ijerph18052519 |
| LeMasters, G. K | Cancer Risk among Firefighters: A Review and Meta-Analysis of 32 Studies | 2006 | https://doi.org/10.1097/01.jom.0000246229.68697.90 |
| Takkouche, B | Risk of Cancer among Hairdressers and Related Workers: A Meta-Analysis | 2009 | https://doi.org/10.1093/ije/dyp283 |
| **Meta-analysis of studies with exposure before 1995 (11)** | | | |
| Acquavella, J | Cancer among Farmers: A Meta-Analysis | 1998 | https://doi.org/10.1016/S1047-2797(97)00120-8 |
| Alif, S. M | Cancer and Mortality in Coal Mine Workers: A Systematic Review and Meta-Analysis | 2022 | https://doi.org/10.1136/oemed-2021-107498 |
| Boffetta, P | Cancer Risk from Occupational and Environmental Exposure to Polycyclic Aromatic Hydrocarbons | 1997 | https://doi.org/10.1023/A:1018465507029 |
| Chen, R | A Meta-Analysis of Mortality among Workers Exposed to Organic Solvents | 1996 | https://doi.org/10.1093/occmed/46.5.337 |
| Collins, J. J | Review and Meta-Analysis of Studies of Acrylonitrile Workers | 1998 | https://www.sjweh.fi/article/314 |
| Fu, H | Cancer and Occupational Exposure to Inorganic Lead Compounds: A Meta-Analysis of Published Data | 1995 | https://doi.org/10.1136/oem.52.2.73 |
| Gaertner, R. R. W | Risk of Bladder Cancer in Foundry Workers: A Meta-Analysis | 2002 | https://doi.org/10.1136/oem.59.10.655 |
| Greenberg, R. S | A Meta-Analysis of Cohort Studies Describing Mortality and Cancer Incidence among Chemical Workers in the United States and Western Europe | 2001 | https://doi.org/10.1097/00001648-200111000-00023 |
| Magnani, C | Italian Pool of Asbestos Workers Cohorts: Asbestos Related Mortality by Industrial Sector and Cumulative Exposure | 2020 | https://doi.org/10.4415/ANN_20_03_07 |
| Tolbert, P. E | [Oils and cancer] | 1997 | https://doi.org/10.1023/A:1018409422050 |
| Wong, O | Critical Review of Cancer Epidemiology in the Petroleum Industry, with a Meta-Analysis of a Combined Database of More than 350,000 Workers | 2010 | https://doi.org/10.1006/rtph.2000.1410 |
| **Not specifically focused on occupational carcinogens (24)** | | | |
| Abbas, N. F | Uncovering the Epidemiology of Bladder Cancer in the Arab World: A Review of Risk Factors, Molecular Mechanisms, and Clinical Features | 2024 | https://doi.org/10.1016/j.ajur.2023.10.001 |
| Andersen, A | Work-Related Cancer in the Nordic Countries | 1999 | https://www.sjweh.fi/article/449 |
| Boffetta, P | Occupation and Bladder Cancer among Men in Western Europe | 2003 | https://doi.org/10.1023/b:caco.0000007962.19066.9c |
| Boffetta, P | Epidemiology of Environmental and Occupational Cancer | 2004 | https://doi.org/10.1038/sj.onc.1207715 |
| Burger, M | Epidemiology and Risk Factors of Urothelial Bladder Cancer | 2013 | https://doi.org/10.1016/j.eururo.2012.07.033 |
| Bouchardy,C | Cancer risk by occupation and socioeconomic group among men -: a study by The Association of Swiss Cancer Registries-Web of Science Core Collection | 2002 | https://www.webofscience.com/wos/woscc/full-record/WOS:000174070200001 |
| Cani, M | How Does Environmental and Occupational Exposure Contribute to Carcinogenesis in Genitourinary and Lung Cancers? | 2023 | https://doi.org/10.3390/cancers15102836 |
| Cumberbatch, M. G. K | Contemporary Occupational Carcinogen Exposure and Bladder Cancer: A Systematic Review and Meta-Analysis | 2015 | https://doi.org/10.1001/jamaoncol.2015.3209. |
| Cumberbatch, M. G. K | The Contemporary Landscape of Occupational Bladder Cancer within the United Kingdom: A Meta-Analysis of Risks over the Last 80 Years | 2017 | https://doi.org/10.1111/bju.13561 |
| Cumberbatch, M. G. K | Epidemiology of Bladder Cancer: A Systematic Review and Contemporary Update of Risk Factors in 2018 | 2018 | https://doi.org/10.1016/j.eururo.2018.09.001 |
| Cumberbatch, M. G. K | Epidemiology, Aetiology and Screening of Bladder Cancer | 2019 | https://doi.org/10.21037/tau.2018.09.11 |
| Ferrís, J | Factores de riesgo constitucionales y ocupacionales asociados al cáncer vesical | 2013 | https://doi.org/10.1016/j.acuro.2013.01.001 |
| Golka, K | Occupational Exposure and Urological Cancer | 2004 | https://doi.org/10.1007/s00345-003-0377-5 |
| Hashim, D | Occupational and Environmental Exposures and Cancers in Developing Countries | 2014 | https://doi.org/10.1016/j.aogh.2014.10.002 |
| Hosseini, B | Occupational Exposure to Carcinogens and Occupational Epidemiological Cancer Studies in Iran: A Review | 2021 | https://doi.org/10.3390/cancers13143581 |
| Jubber, I | Epidemiology of Bladder Cancer in 2023: A Systematic Review of Risk Factors | 2023 | https://doi.org/10.1016/j.eururo.2023.03.029 |
| Kogevinas, M | Occupation and Bladder Cancer among Men in Western Europe | 2003 | https://doi.org/10.1023/b:caco.0000007962.19066.9c |
| Marant Micallef, C | Occupational Exposures and Cancer: A Review of Agents and Relative Risk Estimates | 2018 | https://doi.org/10.1136/oemed-2017-104858 |
| Olfert, S. M | An Updated Review of the Literature: Risk Factors for Bladder Cancer with Focus on Occupational Exposures | 2006 | https://doi.org/10.1097/01.smj.0000247266.10393.72 |
| Park, J | Occupational Reproductive Function Abnormalities and Bladder Cancer in Korea | 2010 | https://doi.org/10.3346/jkms.2010.25.S.S41 |
| Reulen, R. C | A Meta-Analysis on the Association between Bladder Cancer and Occupation | 2008 | https://doi.org/10.1080/03008880802325192 |
| Stojanovic, J | Occupational Exposures and Genetic Susceptibility to Urinary Tract Cancers: A Systematic Review and Meta-Analysis | 2018 | https://doi.org/10.1097/CEJ.0000000000000364 |
| Teglia, F | Occupational Cancers among Employed Women: A Narrative Review | 2023 | https://doi.org/10.3390/cancers15041334 |
| Zaroushani, V | Effect of Occupational Risk Factors in Cancer Incidence in Iran: A Systematic Review | 2021 | https://doi.org/10.18502/ijpho.v11i3.6568 |
